# Prevalence of Panton-Valentine Leukocidin (PVL) and Antimicrobial Resistance in Community-Acquired Clinical *Staphylococcus aureus* in an Urban Gambian Hospital: A 11-Year Period Retrospective Pilot Study

**DOI:** 10.3389/fcimb.2019.00170

**Published:** 2019-05-22

**Authors:** Saffiatou Darboe, Sarah Dobreniecki, Sheikh Jarju, Mamadou Jallow, Nuredin Ibrahim Mohammed, Miriam Wathuo, Buntung Ceesay, Sam Tweed, Robindra Basu Roy, Uduak Okomo, Brenda Kwambana-Adams, Martin Antonio, Richard S. Bradbury, Thushan I. de Silva, Karen Forrest, Anna Roca, Bolarinde Joseph Lawal, Davis Nwakanma, Ousman Secka

**Affiliations:** ^1^Medical Research Council Unit The Gambia at London School of Hygiene & Tropical Medicine, Banjul, Gambia; ^2^Environmental Protection Agency, Washington, DC, United States; ^3^The School of Medicine, Medical Sciences and Nutrition, University of Aberdeen, Aberdeen, United Kingdom; ^4^London School of Hygiene and Tropical Medicine, London, United Kingdom; ^5^School of Health, Medical and Applied Sciences, Central Queensland University, Rockhampton, QLD, Australia

**Keywords:** Panton-Valentine leukocidin, *Staphylococcus aureus*, community-acquired, antimicrobial resistance, The Gambia

## Abstract

**Background:** *Staphylococcus aureus* is a major human pathogen. Panton-Valentine leukocidin (PVL) is a virulence factor produced by some strains that causes leukocyte lysis and tissue necrosis. PVL-associated *S. aureus* (PVL-SA) predominantly causes skin and soft-tissue infections (SSTIs) but can also cause invasive infections such as necrotizing pneumonia. It is carried by both community-associated methicillin susceptible *S. aureus* (CA-MSSA) and methicillin resistant *S. aureus* (CA-MRSA). This study aims to determine the prevalence of PVL-SA among patients seen at an urban Gambian hospital and associated antibiotic resistance.

**Methods:** Archived clinical *S. aureus* (70 invasive bacteraemia and 223 non-invasive SSTIs) from 293 patients were retrieved as well as relevant data from clinical records where available. Antibiotic susceptibility was assessed using disc diffusion according to Clinical Laboratory Standards Institute (CLSI) guidelines. Genomic DNA was extracted and the presence of lukF and lukS PVL genes was detected by conventional gel-based PCR.

**Result:** PVL-SA strains accounted for 61.4% (180/293) of *S. aureus* isolates. PVL prevalence was high in both Gambian bacteraemia and SSTIs *S. aureus* strains. Antimicrobial resistance was low and included chloramphenicol (4.8%), cefoxitin (2.4%), ciprofloxacin (3.8%), erythromycin (8.9%), gentamicin (5.5%) penicillin (92.5%), tetracycline (41.0%), and sulfamethoxazole-trimethoprim (24.2%). There was no association of PVL with antimicrobial resistance.

**Conclusion:** PVL expression is high among clinical *S. aureus* strains among Gambian patients. Reporting of PVL-SA clinical infections is necessary to enable the monitoring of the clinical impact of these strains in the population and guide prevention of the spread of virulent PVL-positive CA-MRSA strains.

**SUMMARY**

*Staphylococcus aureus* (*S. aureus*) is a major human pathogen with several virulence factors. We performed a retrospective analysis to investigate the prevalence of one such virulence factor (PVL) amongst clinical *S. aureus* samples. We found a high prevalence in our setting but antimicrobial resistance including methicillin resistance was low.

## Introduction

*Staphylococcus aureus* (*S. aureus*) is a major human pathogen causing infections ranging from mild skin and soft-tissue infections to life-threatening sepsis, and implicated in both community acquired (CA) and healthcare-associated (HA) infections with significant morbidity and mortality (Boyle-Vavra and Daum, 2007; Breurec et al., 2011a; Ozekinci et al., 2014). A major predisposing factor for infection is surface colonization; *S. aureus* colonization of the skin and mucus membranes is estimated in 30% of healthy individuals while a further 30% are transient carriers (Chambers and Deleo, 2009; Shallcross et al., 2013). The remarkable capacity to acquire methicillin resistance first in hospitals and more recently in community strains adds to the success of *S. aureus* as a pathogen due to widespread use of β-lactams in the treatment of infection (Cosgrove et al., 2003).

The pathogenesis of *S. aureus* is mediated by several cell surface and secreted virulence factors. One such virulence factor, Panton-Valentine leukocidin (PVL) is a two-component toxin, that induces pore formation in the leukocyte cell membrane complement receptors (Rasigade et al., 2010). These two genes (LukS-PV and LukF-PV) encode two proteins which are co-transcribed and secreted separately to form a complete heptavalent leukocidin (Boyle-Vavra and Daum, 2007; Rasigade et al., 2010). Although produced by <5% of *S. aureus* strains, PVL is detected in large percentages of isolates that cause necrotic skin lesions and severe necrotizing pneumonia (Lina et al., 1999; Labandeira-Rey et al., 2007; Shallcross et al., 2013). PVL-associated *S. aureus* (PVL-SA) infection is commonly associated with community-acquired methicillin resistant *S. aureus* (CA-MRSA) (Asiimwe et al., 2017), but outbreaks due to PVL-pos methicillin-susceptible *S. aureus* (MSSA) strains have been reported in close community settings (Boubaker et al., 2004; Rasigade et al., 2010). However, the potential risk of spread to hospital is considered a significant public health concern, as establishment of a PVL positive clonal lineage of HA-MRSA could rapidly lead to dramatically worse outcomes for HA-MRSA patients (Witte, 2009; Breurec et al., 2011b). There are clonal lineages from which HA-MRSA and CA-MRSA have been reported (Boyle-Vavra and Daum, 2007; Witte, 2009; Breurec et al., 2011b).

Invasive *S. aureus* infections throughout Europe, America and parts of Africa are often caused by strains lacking the PVL gene (Shallcross et al., 2013), however, some studies have described the epidemiological relevance of PVL-positive MSSA as a reservoir for CA-MRSA and a marker of severe infection (Witte, 2009; Rasigade et al., 2010). The higher pathogenic potential and recurrence of CA-MRSA has been attributed to the ability of these organisms to express PVL (Breurec et al., 2011b). However, the role of PVL alone on severity of disease is controversial with studies suggesting additional role of hemolysin β contributing to virulence thus the need for more robust study on hemolysins (Lebughe et al., 2017). Data across West Africa (Senegal, Cameroon, and Ghana) indicate a high prevalence of PVL-positive MSSA in infection, as opposed to North, East and Southern Africa (Breurec et al., 2011a; Egyir et al., 2014; Dekker et al., 2016; Asiimwe et al., 2017) and in the UK (Shallcross et al., 2010).

No data on PVL prevalence has been reported in The Gambia and it is paramount to determine the prevalence of a potential virulence gene cluster in the strains present. This study aims to determine the PVL prevalence and its association with antibiotic resistance in *S. aureus* isolated from clinical strains between 2005 and 2015 in an urban hospital of The Gambia.

## Methods

### Setting and Population

The study was conducted in the Clinical Services Department (CSD) of the Medical Research Council Unit The Gambia (MRCG) at the LSHTM, Fajara which is located in the urban western region of The Gambia and serves the population mainly from this region. The population of the Gambia is 2.1 million (The Gambia Bureau Statistics, 2014) and the hospital has a 42-bed in-patient unit and an outpatients' department seeing more than 50,000 patients annually. It is the only health facility were routine microbiological investigations are carried out for patients in this region. Pathogens of clinical significance were stored in −70°C for potential future research of public health significance.

### Study Design

This was a cross sectional study based on data compiled from records and samples for patients seen at The MRCG clinic during the period January 2005—December 2015 to determine the presence of PVL in Gambian clinical *S. aureus*. All *S. aureus* positive blood cultures from patients with suspected sepsis during the study period were included. SSTI samples from skin related infections such as pus, wounds etc. collected from clinically symptomatic cases within 2 weeks as the invasive samples for culture and antimicrobial testing were included in this study. Isolates were retrieved for patients who had *S. aureus* isolated within 72 h of admission and on day of visit for those from outpatients. Duplicate isolates from the same patient were excluded. Invasive isolates from other sites other than blood culture were also excluded.

### Clinical Data

Clinical records were retrieved for invasive cases and reviewed, where available, to ascertain length of hospital stay, severity of disease and mortality.

### Microbiological Procedures

Two hundred and ninety-three (293) *S. aureus* isolates collected from 293 patients were retrieved from storage at −70°C. Invasive isolates were all from blood (70/293; 24%) and 223 were from non-invasive skin and soft tissue. Isolates were sub cultured on blood agar and incubated overnight at 37°C.

Isolates were identified using a combination of enzymatic and fermentation tests, including catalase, tube coagulase, DNase, and mannitol fermentation.

Antimicrobial susceptibility was done by disc diffusion according to the Clinical Laboratory Standard Institute (CLSI) guidelines (CLSI., 2015) using the following antibiotics: cefoxitin (Fox), chloramphenicol (C), ciprofloxacin (Cip), erythromycin (E), gentamicin (Cn), penicillin (P), sulfamethoxazole-trimethoprim (Sxt), and tetracycline (Te). The cefoxitin disc was used as a surrogate for all penicillinase-stable penicillins and resistance was used to infer *mecA*-mediated methicillin resistance. Where cefoxitin was resistant by disc diffusion, an oxacillin E-test (bioMérieux, Marcy l'Etoile, France) was performed, with an oxacillin MIC ≥4 ug/mL used to define methicillin resistance (CLSI., 2015). The antimicrobial susceptibility testing was carried out using the coagulase positive methicillin susceptible *S. aureus* ATCC 25923 and coagulase negative *Staphylococcus epidermidis* ATCC 12228, respectively for quality control.

### Molecular Typing by Gel Based PCR

Genomic DNA was extracted using QIAamp DNA Mini Kit (QIAGEN, Netherlands). Isolated colonies were suspended in 1X TE buffer to 1.0 McFarland Standard turbidity and suspended cells were then lysed in lysostaphin (Sigma-L7386) at a concentration of 200μg/ml and DNA extraction performed as per the Qiagen protocol. N65 (PVL and γ-hemolysin deficient) (Supersac et al., 1993) and ATCC 49775 (PVL and γ-hemolysin) (Supersac et al., 1998) were used as negative and positive controls, respectively.

The conventional gel-based PCR reaction mix was performed in a 25 μl reaction volume, which included 12.5 μl of 2X Taq master mix (Qiagen, Netherlands), and 2 μl of 10 μM concentration forward and reverse primers. At least 50 ng of extracted genomic DNA was required for each reaction. Primers used for detecting presence of lukF-PV and lukS-PV PVL genes were designed using GenBank sequences (accession numbers AB006796 and L01055). Primers to amplify 16S rDNA have previously been established (De Buyser et al., 1989). The following PCR conditions were used for PVL PCRs: an initial denaturation of 94°C for 3 min followed by 35 cycles of denaturation at 94°C for 1 min, annealing temperatures of 60°C for *lukSPV* and 55°C for *lukFPV*, and an extension of 72°C for 1 min. Final extension was at 72°C for 10 min. Post PCR gel electrophoresis was performed to identify the presence or absence of the above-mentioned genes. The gel percentage used was as per the fragment size of the gene concerned. Ten percentage of the samples were randomly selected and repeated by a different individual for reproducibility and they were found to be reproducible.

### Statistical Analysis

Data were analyzed using STATA version 12.1. Sample characteristics and the prevalence of *S. aureus* by sample type were generated using summary tables. A chi-squared test was used to determine the association between PVL status and sample type. Logistic regression models were fitted to determine the association between PVL status and antimicrobial resistance.

### Ethical Review and Approval

The study received ethical approval from the Joint MRCG @LSHTM/Gambia Government Ethics Committee. Clinical samples were collected for standard clinical management and the results were anonymized for analytical purpose.

## Results

**Figure 1:**
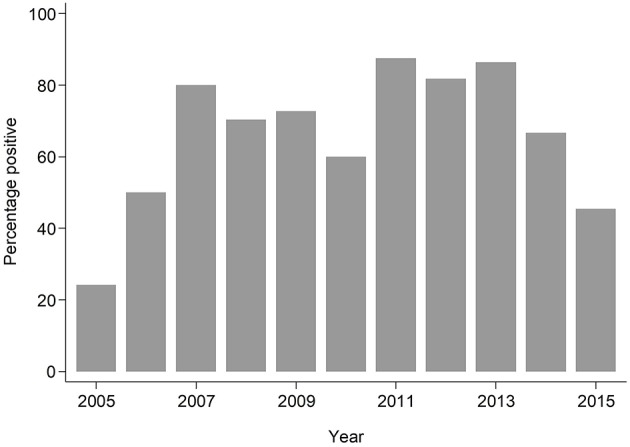
Bar plot showing proportion of PVL positive samples by year.

The median age of individuals in the study was 48 months (IQR 12, 252) (Table 1). The overall prevalence of PVL genes was 180/293; 61.4% (95% CI: 55.6%, 67.0%), with some evidence (*p* = 0.024) of higher prevalence seen in invasive samples 51/70; 72.9% than non-invasive samples 129/223; 57.9% (Table 1). PVL prevalence was consistently high across all the years exceeding 20% (Figure 1).

**Table 1:** Sample characteristics.

**Variable**	**Category**	***n* = 293**	**PVL status**
			**Positive (%)**
Age group	<2 months	33	25 (75.8)
	2–23 months	65	44 (67.7%)
	24–59 months	36	17 (47.2%)
	5–14 years	53	35 (66.0%)
	≥15 years	106	59 (55.7%)
Sex^*^	Female	150	92 (61.3%)
	Male	140	87 (62.1%)
Sample type	Skin and soft tissues	223	129 (57.9%)
	Bacteraemia	70	56 (71.8%)
Year	2005	29	7 (24.1%)
	2006	20	10 (50.0%)
	2007	10	8 (80.0%)
	2008	27	19 (70.4%)
	2009	22	16 (72.7%)
	2010	5	3 (60.0%)
	2011	16	14 (87.5%)
	2012	22	18 (81.8%)
	2013	22	19 (86.4%)
	2014	54	36 (66.7%)
	2015	66	30 (45.5%)

* *Some missing data. Overall PVL prevalence and 95% CI = 180/293 = 61.4% (55.6%, 67.0%)*.

**Figure 2:**
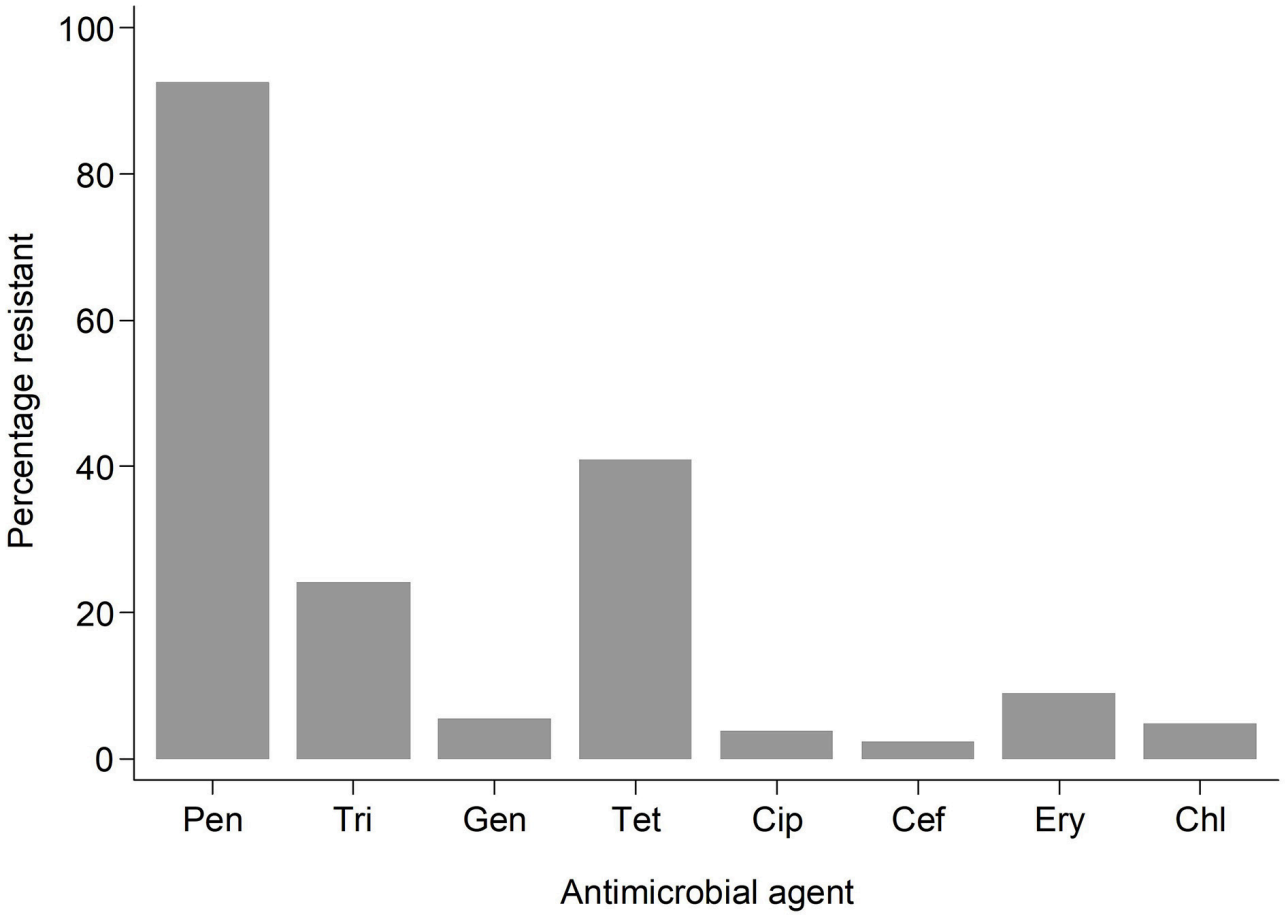
Bar graph showing resistant samples by drug. Pen, Penicillin; Tri, Trimethoprim-sulphamethoxazole; Gen, Gentamicin; Tet, Tetracycline; Cip, Ciprofloxacillin; Cef, Cefoxitin; Ery, Erythromycin; Chl, Chloramphenicol.

**Figure 3:**
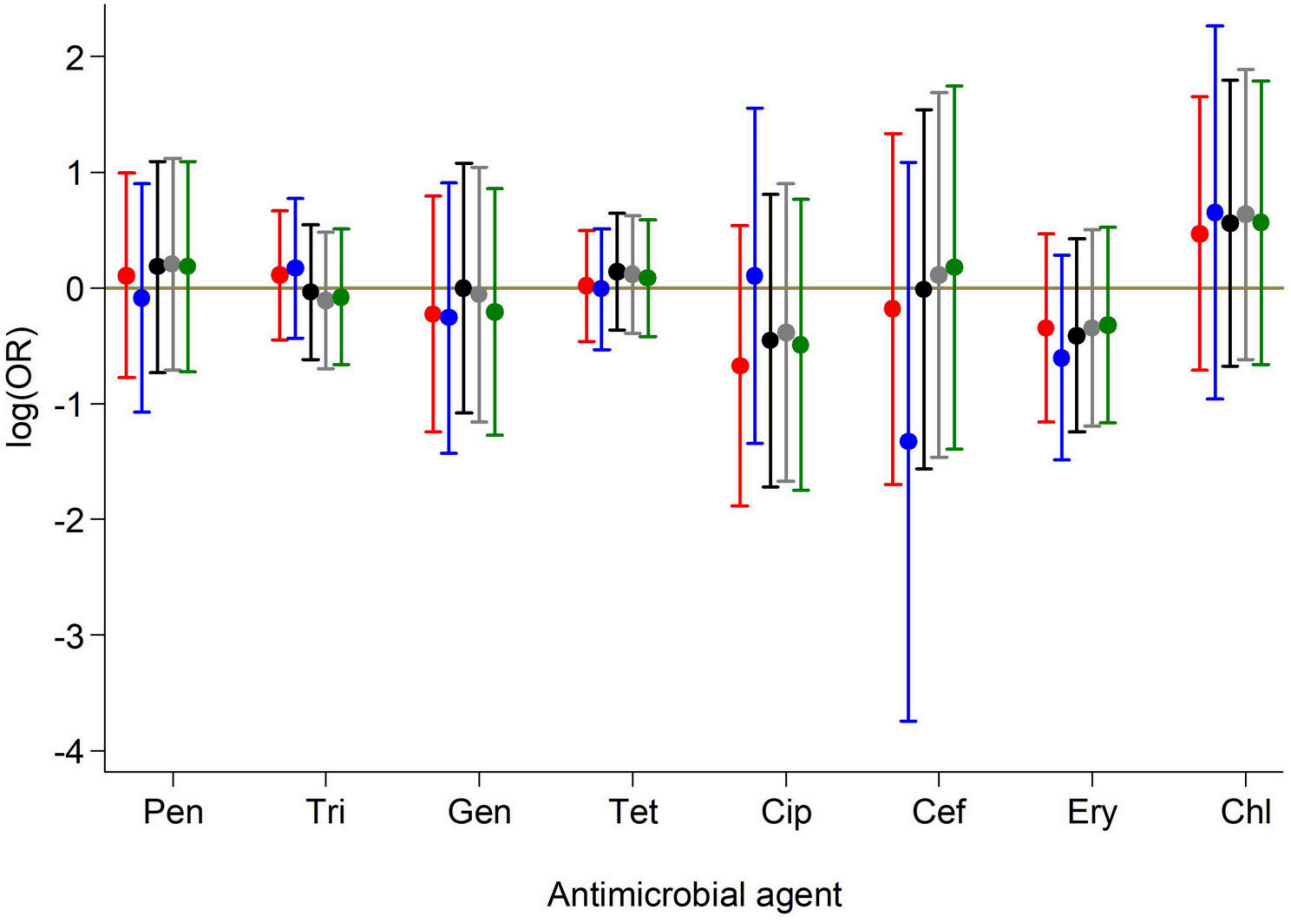
Association between antimicrobial resistance and PVL status. Results from crude and adjusted binary logistic regression models with resistance to each of the antibiotics as the outcome and PVL status as the predictor. ORs and 95% CIs on the log scale for PVL status are presented based on models: 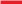 Unadjusted for any covariate; 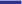 Adjusted for continuous age, sex, year of sample collection; 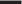 Adjusted for categorical age, sex, year of sample collection; 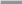 Adjusted for categorical age, sex, year of sample collection, sample type; 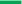 Adjusted for categorical age, sex, year of sample collection (Categorical in two periods), sample type. Pen, Penicillin; Tri, Trimethoprim-sulphamethoxazole; Gen, Gentamicin; Tet, Tetracycline; Cip, Ciprofloxacillin; Cef, Cefoxitin; Ery, Erythromycin; Chl, Chloramphenicol.

Antimicrobial resistance (AMR) to various antimicrobials was analyzed (Figure 2). Resistance to penicillin (penicillinase-labile) (271/293; 92.5%) was high but low for penicillinase-stable penicillins such as methicillin (7/293; 2.4%). Association between AMR and with PVL status was also analyzed (Figure 3). Although methicillin resistant *S. aureus* (MRSA) rates were low, no difference was found in the rate of PVL prevalence in 4/7 (57.1%) of MRSA cases compared with the MSSA 176/286 (61.5%). The range of oxacillin MICs in the seven MRSA isolates was universally high, between ≥9 and ≥23 μg/mL, denoting significant resistance to penicillinase-stable penicillins. There was no evidence of association between antimicrobial resistance and PVL status. Although clinical data was only available for a subset of cases of bacteraemia (29/70), length of hospital stay, mortality or gender were not associated with PVL status (*p* = 0.4).

## Discussion

This study raises awareness of the important virulent gene cluster present in *S. aureus* causing disease in our setting. This study confirms high PVL prevalence in *S. aureus* causing disease and contributes to current knowledge in prevalence of PVL in The Gambia where *S. aureus* is currently the most prevalent pathogen causing bacteraemia (Darboe et al., 2019) (accepted for publication CID).

The prevalence of PVL (72.9%) in invasive disease was unusually high but similar to that found in Ghana (75%) (Dekker et al., 2016), and greater than reported in the neighboring country of Senegal (47%), elsewhere in Africa and across the globe (Breurec et al., 2011a; Shallcross et al., 2013). Prevalence in SSTI however was similar to other West African countries including Senegal (Breurec et al., 2011a). The study also confirms that PVL prevalence was high across all age groups.

Antibiotic susceptibility was similar to what was described for *S. aureus* causing infection in other parts Africa where multidrug resistance continues to be high for penicillin, sulfamethoxazole-trimethoprim and tetracycline (Schaumburg et al., 2014; Dekker et al., 2016), reflecting the frequent use of these drugs in the community. MRSA continues to occur at low prevalence (2.6% of infections), similar to what was described in Ghana and neighboring Senegal (Dekker et al., 2016) as opposed to what is described for other parts of Africa (Breurec et al., 2011a). Although resistance to sulfamethoxazole-trimethoprim was high, no association was found between PVL expression in contrast to what was found in Saudi Arabia (Bazzi et al., 2015), Nigeria (Ayepola et al., 2018), and in Gabon (Kraef et al., 2015).

In this study, we did not find evidence for an association between PVL status and length of hospital stay in patients admitted for bacteraemia supporting the evidence that PVL-positive strains does not predict poor outcome in these patients (Shallcross et al., 2013). This may highlight the fact that PVL status has no impact on pathogenicity in invasive disease as the presence of the gene does not infer expression (Ayepola et al., 2018). We did not compare clinical outcome and PVL status in SSTIs where strong association is suggested in many studies (Shallcross et al., 2010, 2013; Ozekinci et al., 2014). Other studies have linked younger age, hospitalization and severity of disease as risk factors for PVL (Muttaiyah et al., 2010). In a study in Taiwan, PVL positive MSSA infection was linked to longer duration of fever (Chiu et al., 2012). There is also strong evidence that PVL is associated with disease and less with colonization; with certain lineages and sequence types (ST) such as ST152, ST121, and ST5 more likely to harbor it (Breurec et al., 2011a; Shallcross et al., 2013; Egyir et al., 2014; Ayepola et al., 2015). The findings of various studies are summarized in Appendix 1.

There are several limitations of this study. The first and most important being the inclusion of only one hospital and sampling based on availability of isolate. This may not be representative of the entire country. Another limitation is that, being a retrospective study, it was not possible to inform the attending physicians of the results to impact on care. Although patients were mostly directly from the community, occasional admissions within 3 days were all assumed to be community acquired and risk factors of disease were not ascertained. Length of hospital stay was not analyzed for many patients due to lack of clinical records. Other clinical outcomes such as prognosis and mortality were not assessed due to limited data. In addition, SSTIs were not categorized into the various sample types and clinical outcome was not assessed. Contact tracing among family members and sequencing the strains for genetic relatedness would also have been useful. PVL phenotypic expression was not confirmed to compare rate detected against that expressed. Future research should consider these and other typing for lineages based on sequence types and staphylococcal protein A (spa) to provide more information into *S. aureus* lineages circulating especially for strains causing bacteraemia and meningitis.

Notwithstanding, this paper has filled a knowledge gap for this pathogen. Our sample size was reasonably large and longitudinal as well as being the first published literature to determine the presence of such an important virulence gene in a setting that has low prevalence of MRSA. We have demonstrated that contrary to the rarity of PVL in invasive disease reported (Shallcross et al., 2013), we have a high prevalence, concordant with rates found in neighboring Senegal and Ghana (Breurec et al., 2011a; Dekker et al., 2016). The CA-MRSA in the Senegal/Gambia region of West Africa may be a potential emerging public health concern as they may serve as reservoir for the potential spread of virulent strains into healthcare. Hence this requires close monitoring to inform treatment options and the development of strategies to control the spread of these difficult to treat infections in the region.

In conclusion, PVL prevalence was high in both Gambian invasive and SSTIs *S. aureus* strains and no association was found with antimicrobial resistance. These findings warrant further investigation into the diversity of *S. aureus* lineages as this could be an important finding to assess the likely reservoir for PVL-positive CA-MRSA in our setting.

## Data Availability

The datasets generated for this study are available on request to the corresponding author.

## Author Contributions

SD, SaD, BL, DN, and OS conceived the idea and designed the study. SD, SaD, SJ, MJ, and BC performed the laboratory analysis and ST correlated clinical data. MW, NM, MJ, and SD analyzed the data prepared the tables and figures. SD, SaD, SJ, MW, and RB wrote the manuscript. UO, BK-A, MA, TdS, KF, RSB, AR, DN, and OS critically revised the manuscript. All authors reviewed the manuscript.

### Conflict of Interest Statement

The views expressed in this article are those of the author, SaD, and do not necessarily represent the policies or positions of the Environmental Protection Agency or the United States. RSB is co-authoring this manuscript in his personal capacity and in his role as an adjunct academic at Central Queensland University. The remaining authors declare that the research was conducted in the absence of any commercial or financial relationships that could be construed as a potential conflict of interest.
