# Supplementary material for: Prevalence of Panton-Valentine Leukocidin (PVL) and Antimicrobial Resistance in Community-Acquired Clinical Staphylococcus aureus in an Urban Gambian Hospital: A 11-Year Period Retrospective Pilot Study
Source: Front Cell Infect Microbiol. 2019 May 22;9:170. doi: 10.3389/fcimb.2019.00170 (PMC6540874; doi:10.3389/fcimb.2019.00170)
Supplement: Supplementary file 1 [file Table_1.docx]

Supplementary data

**Appendix 1:**

**Table 2; Summary of similar studies reporting PVL prevalence**

| **Study title** | **Sampling** | **Study period** | **Number** | **Results** | **indication** | **Country** | **Region** |
| --- | --- | --- | --- | --- | --- | --- | --- |
|  |  |  |  |  |  |  |  |
| Molecular Characterisation and Antimicrobial Susceptibility of Staphylococcus aureus from Clinical Infection and Asymptomatic Carriers in Southwest Nigeria | Clinical infection (invasive and non-invasive) and asymptomatic carriage; from 8 Healthcare centres | 2010 and 2011 | Two hundred and seventeen (217) clinical and Seventy-three (73) carriage MSSA and MRSA | - PVL positive higher for clinical 174/217; 80.2% than 39/73; 53.4% carriage. - MRSA 2.4%,   MDR (P, Sxt, Te) in both clinical and carriage samples. Aminoglycoside resistant was higher for clinical isolates whilst quinolone and tetracycline were higher in carriage.   - Infection isolates were of different lineages, 14 spa types associated with infection | - PVL higher in clinical isolates indicating association with disease - evidence of associated with sulphonamide- trimethoprim and penicillin resistance - Infection and carriage lineages were different | Nigeria | Sub-Saharan Africa |
| Antibiotic resistance and clonal diversity of invasive Staphylococcus aureus in the rural Ashanti Region, Ghana | Routine clinical care for bacteraemia (invasive), all age groups in 2 rural hospitals | May 2007 to August 2012 | Fifty-six (56) MSSA and MRSA | - PVL positive MSSA 42/56; 75% - MRSA 1.8%, MDR to P, Sxt, Te, - ST121, ST152, ST15 likely to be PVL pos | - PVL associated with certain STs - Diverse lineages found | Ghana | Sub-Saharan Africa |
| Prevalence of Panton-Valentine leukocidin-positive methicillin-susceptible Staphylococcus aureus infections in a Saudi Arabian hospital. | Routine clinical care for hospitalised and non-hospitalised, all age groups, invasive and non-invasive samples | January to December 2013 | Ninety-three (93) MSSA | - PVL-pos   28/93; 30%   - 39.2% of PVL pos strains resistant ot sxt - MDR P, Sxt, macrolides, Te | - PVL associated with SXT resistance, - Younger age a risk factor, | Saudi Arabia | Middle East |
| Panton-valentine leukocidin in community and hospital-acquired staphylococcus aureus strains. | Clinical samples from hospital and community in from 0 to 79 years | 2012 | Seventy (70)’  38 MRSA and 32 MSSA | - Overall, PVL-pos 12/70; 17% - MSSA PVL-pos 8/32; 25% - MRSA PVL 4/38; 10.5% - PVL-pos for 8/36; 22% of community strains and 4/34; 12% of hospital | - High rates of PVL found in both community and hospital strains | Turkey | Europe |
| Panton-valentine leucocidin associated staphylococcal disease: A cross-sectional at a London hospital, England. | Clinical samples from hospital and community | 15 July to September 2007 | Three hundred and ninety (390), MSSA and MRSA | - Overall, PVL-pos 38/390; 9.7%, - PVL-pos MRSA 3/66; 4.5% - PVL pos MSSA 35/324; 10.8% | - PVL-SA more common in community than hospital and was more in MSSA - Male gender SSTI more associated with | England | Europe |

**Appendix 2:**

**Table 3; Logistic regression results**

| **Model** | **Antibiotic (outcome)** | **Log OR (95% CI)** | **p-value** |
| --- | --- | --- | --- |
| Crude | Penicillin | 0.11 (-0.78, 0.99) | 0.815 |
|  | Trimethoprim | 0.11 (-0.45, 0.66) | 0.699 |
|  | Gentamicin | -0.23 (-1.24, 0.79) | 0.663 |
|  | Tetracycline | 0.02 (-0.46, 0.5) | 0.946 |
|  | Ciprofloxacin | -0.67 (-1.88, 0.54) | 0.275 |
|  | Cefoxitin | -0.18 (-1.7, -0.33) | 0.814 |
|  | Erythromycin | -0.35 (-1.16, 0.46) | 0.404 |
|  | Chloramphenicol | 0.47 (-0.71, -0.65) | 0.425 |
| Adjusted (sex, age in months, year) | Penicilin | -0.09 (-1.08, 0.9) | 0.394 |
|  | Trimethoprim | 0.17 (-0.43, 0.77) | 0.181 |
|  | Gentamicin | -0.26 (-1.43, 0.91) | 0.329 |
|  | Tetracycline | -0.01 (-0.53, 0.51) | 0.406 |
|  | Ciprofloxacin | 0.1 (-1.34, -0.55) | 0.155 |
|  | Cefoxitin | -1.33 (-3.74, -0.08) | 0.07 |
|  | Erythromycin | -0.6 (-1.49, 0.28) | 0.409 |
|  | Chloramphenicol | 0.65 (-0.96, -0.26) | 0.516 |
| Adjusted (age linear trend, sex, year) | Penicilin | 0.18 (-0.73, -0.09) | 0.356 |
|  | Trimethoprim | -0.04 (-0.62, 0.54) | 0.07 |
|  | Gentamicin | -0 (-1.08, -0.08) | 0.294 |
|  | Tetracycline | 0.14 (-0.36, 0.65) | 0.108 |
|  | Ciprofloxacin | -0.46 (-1.72, 0.81) | 0.476 |
|  | Cefoxitin | -0.01 (-1.57, -0.54) | 0.902 |
|  | Erythromycin | -0.41 (-1.25, 0.42) | 0.361 |
|  | Chloramphenicol | 0.56 (-0.68, -0.8) | 0.627 |
| Adjusted (age linear trend, sex, year, sample type) | Penicilin | 0.2 (-0.71, -0.12) | 0.424 |
|  | Trimethoprim | -0.11 (-0.7, 0.48) | 0.02 |
|  | Gentamicin | -0.06 (-1.16, -0.04) | 0.37 |
|  | Tetracycline | 0.12 (-0.39, 0.62) | 0.126 |
|  | Ciprofloxacin | -0.39 (-1.67, 0.9) | 0.464 |
|  | Cefoxitin | 0.11 (-1.47, -0.69) | 0.929 |
|  | Erythromycin | -0.35 (-1.19, 0.5) | 0.226 |
|  | Chloramphenicol | 0.63 (-0.62, -0.89) | 0.619 |
| Adjusted (grouped year, age, sex, sample type) | Penicilin | 0.18 (-0.73, -0.09) | 0.443 |
|  | Trimethoprim | -0.08 (-0.66, 0.51) | 0.044 |
|  | Gentamicin | -0.21 (-1.27, 0.86) | 0.413 |
|  | Tetracycline | 0.08 (-0.42, 0.59) | 0.135 |
|  | Ciprofloxacin | -0.49 (-1.75, 0.77) | 0.241 |
|  | Cefoxitin | 0.18 (-1.39, -0.74) | 0.977 |
|  | Erythromycin | -0.32 (-1.16, 0.52) | 0.33 |
|  | Chloramphenicol | 0.56 (-0.66, -0.79) | 0.461 |
